# Historical evolution and processing mechanism of ‘nine steaming and nine drying’ of traditional Chinese medicine preparation

**DOI:** 10.1080/13880209.2024.2354345

**Published:** 2024-05-16

**Authors:** Yong-kang Li, Zhi Chen, Chao Zhang

**Affiliations:** College of Pharmacy, Shandong University of TCM, Jinan, China

**Keywords:** Drug properties, tonifying effect, chemical composition, pharmacological activity

## Abstract

**Context:**

Nine steaming and nine drying is a traditional Chinese medicine (TCM) processing method and it is widely used for processing tonifying herbs. Modern research reveals that the repeated steaming and drying process varies the composition and clinical efficacy of TCM.

**Objective:**

This paper analyzes and explores the historical evolution, research progress, development strategies, and problems encountered in the nine steaming and nine drying process so as to provide a reasonable explanation for this method.

**Methods:**

English and Chinese literature from 1986 to 2023 was collected from databases including Web of Science, PubMed, Elsevier, Chinese Pharmacopoeia 2020 (CP), and CNKI (Chinese). Nine steaming and nine drying, processing, TCM and pharmacological activity were used as the key words.

**Results:**

Nine steaming and nine drying has undergone thousands of years of clinical practice. Under specific processing conditions of nine steaming and nine drying, the ingredients of the TCM have significant changes, which in turn altered clinical applications.

**Conclusions:**

This review provides sufficient evidence to prove the rationality and scientific value of nine steaming and nine drying and puts forward a development direction for future research.

## Introduction

The nine steaming and nine drying method reflects basic theoretical knowledge of TCM that has been enriched over thousands of years in China and embodies wisdom of ancient Chinese as well as medication characteristics of TCM (Zhou et al. 2016; Meng et al. 2017; Fan B et al. 2020). This method is mainly used to prepare tonifying TCM in modern times. The herbs processed by nine steaming and nine drying mainly include the roots, rhizomes, fruits, and seeds of plants, and this method mainly rectifies drug properties or increases the content of active ingredients and decreases the content of toxic ingredients for better clinical effects (Teng et al. 2022). Generally speaking, medicinal materials should be fully steamed by water vapor, which sometimes involves the addition of auxiliary material such as rice wine, and then spread out and adequately dried in the sun (Cheng et al. 2021). As the number of steaming and drying times increases, the colors, gloss, and appearance of medicinal materials will change greatly. This also indirectly indicates that the chemical composition of the TCM has changed, and then affects the efficacy. Modern pharmacological experiments have shown that TCM prepared by nine steaming and nine drying has extensive pharmacological activity and deserves further research (Liu and Si 2018; Wu et al. 2018).

## Historical evolution

The historical evolution of the processing method is closely related to the ancient development of Chinese herbal medicine preparation. Nine steaming and nine drying started from the Northern and Southern Dynasties, and involved 3 kinds of TCM during the Tang Dynasty, 11 kinds of TCM during the Song Dynasty, 2 kinds of TCM during the Yuan Dynasty, 12 kinds of TCM during the Ming Dynasty, 19 kinds of TCM during the Qing Dynasty and only 2 kinds of TCM during the period of the Republic of China. During and before the Tang and Song Dynasties, most medicinal materials were purely steamed without auxiliary material. Since the Ming and Qing Dynasties, types of medicinal material processing by nine steaming and nine drying increased and some drugs were steamed with multiple auxiliary materials. The historical evolution of four typical TCM processed by nine steaming and nine drying is shown in Table 1.

**Table 1.** Historical evolution of four typical TCM processed by nine steaming and nine drying.

Variety of medicinal materials	Dynasty	Ancient medical literature	The record of ‘nine steaming nine drying’
*Sesamum indicum* L. (Pedaliaceae) seed (Huma)	Southern and Northern Dynasties	Annotations on the Classic of Materia Medica	Nine steaming, nine sun-exposing, frying, and pounding, if not steamed well, it can cause hair loss
	Tang Dynasty	Beiji Qianjin Yaofang	Steam thirty times, bake and grind
	Tang Dynasty	Newly Revised Materia Medica	Nine steamed and nine exposed, stir fried, mashed
	Song Dynasty	Bencao Tujing	To eat its fruit, it should be steamed, exposed to sun nine times, fried, and mashed
	Ming Dynasty	Primitive Materia Medica	Take black sesame, steam and sun nine times, stir fry and mash before taking
	Qing Dynasty	Materia Medica Description	Nine steams and nine exposures, then sunlight three times combined with Maoshan *Atractylodes macrocephala* and milk, can strengthen the spleen, dry dampness, and replenish qi
	Qing Dynasty	Wo Ling Materia Medica	Flax is processed using the nine steaming and nine drying method, and prepared into a pill with jujube paste. After taking it, white hair returns to black
*Polygonatum sibiricum* Delar. ex Redoute. (Liliaceae) root (Huangjing)	Tang Dynasty	Herbal Food Medicine	Nine steaming and nine drying
	Song Dynasty	Rihuazi Materia Medica	Nine steaming and nine drying, can nourish the skin after use
	Five Dynasties	Shu ben cao	The best medicinal effect is to take it with nine steamed and nine dried by sunshine, and if it is dried in the shade, it may cause decay
	Ming Dynasty	Herbal for Relief of Famines	Processed using the nine steams and nine storms method, if the degree of processing is not sufficient, it can irritate the throat
	Ming Dynasty	Bencao Mengquan	Nine steamed and nine exposed, can replace grain
	Ming Dynasty	Compendium of Materia Medica	Huangjing and Manjingz steamed nine times and sun dried nine times together
*Rehmannia glutinosa*(Gaert.) Libosch. ex Fisch. et Mey. (Scrophulariaceae) tuberous root (Dihuang)	Tang Dynasty	Supplement to Invaluable Prescriptions for Ready Reference	The ancient method required nine steaming and nine sun drying. Now, it is observed that the liquid is completely absorbed and the color turns black
	Song Dynasty	Bencao Tujing	Dihuang should be processed until its appearance appears black and shiny, with a high sweetness
	Song Dynasty	Classified Materia Medica	Dried *Rehmannia glutinosa* after steaming, mixed with *Rehmannia* liquid, soaked, dried, steamed
	Yuan Dynasty	Decoction and Material Medica	After steaming *Rehmannia glutinosa* nine times, it can replenish the kidney’s vital energy
	Ming Dynasty	Herbal for Relief of Famines	Nine steamed and nine sun dried
	Ming Dynasty	Compendium of Materia Medica	Steamed and sun dried nine times with *Amomum villosum* and rice wine
	Qing Dynasty	Materia Medica Description	*Rehmannia glutinosa* does not achieve its effectiveness through a single process
	Republic of China	Identification of Additional Counterfeit Drug Articles	*Rehmannia glutinosa* needs to be processed through nine steaming and nine sun drying processes. The transparent black color has good quality, but if the center is slightly yellow, the quality is poor
*Fallopia multiflora* (Thunb.) Harald. (Polygonaceae) tuberous root (Heshouwu)	Song Dynasty	Peaceful Holy Benevolence Formulae	Nine steaming and nine drying, roasting and mashing
	Song Dynasty	Bencao Tujing	Use after nine steaming and nine sun exposure
	Ming Dynasty	Ben cao pin hui jing yao	Steamed together with dates or black beans
	Ming Dynasty	Compendium of Materia Medica	Use after nine steaming and nine sun exposure
	Ming Dynasty	Bencaohuiyan	*Polygonum multiflorum* needs nine steaming and nine sun exposure to reduce its toxicity.
	Qing Dynasty	Supplements to Compendim of Materia Medica	Processing *Polygonum multiflorum* with black beans plays a role in guiding medicine into the kidney meridian

### The northern and southern dynasties

The predecessor of nine steaming and nine drying was first seen in Leigong Treatise on the preparation in Liu Song period of Southern and Northern dynasties (Lei X 1986). In this book, TCM processed by steaming only is recorded. *Sesamum indicum* L. (Pedaliaceae) seed (Huma) is an ancient and important oilseed crop. Moreover, it has been utilized for medicinal purposes, especially in the Eastern world (Hahm et al. 2009; El-Roby et al. 2020). Annotations on the Classic of Materia Medica describes processing of Huma by nine steaming and nine drying, namely ‘steam and expose it to the sun for nine times, then decoct and smash it. If it’s not well steamed, it may lead to hair loss’. This is the first appearance of nine steaming and nine drying in ancient Chinese medical literature. It points out emphatically that Huma is processed by nine steaming and nine drying and the processed product shows an effect of health care; raw Huma can cause baldness (Tao HJ 1994). It showed that the ancients had begun to explore the method of nine steaming and nine drying and had accumulated some experience, and reached the conclusion that the beneficial effect could be enhanced.

### The Tang and Song dynasties

Since the Tang and Song Dynasties, literature about nine steaming and nine drying became increasingly rich and the number of TCM processed by this method increased to 14, including *Polygonatum sibiricum* Delar. ex Redoute. (Liliaceae) root (Huangjing), *Rehmannia glutinosa* (Gaert.) Libosch. ex Fisch. et Mey. (Scrophulariaceae) tuberous root (Dihuang), *Siegesbeckia orientalis* Linn. (Compositae) herba (Xixian), *Fallopia multiflora* (Thunb.) Harald. (Polygonaceae) tuberous root (Heshouwu), *Rheum officinale* Baill. (Polygonaceae) root (Dahuang) etc. During this period, ancients identified irritation of raw Huangjing. Clinically, when patients took raw Huangjing, their throats were usually irritated, resulting in numb mouths and tongues; when people’s skin touched raw Huangjing or its juice, it could lead to pruritus; If exposed for a long time, it will sting one’s eyes. As a result, nine steaming and nine drying not only removed irritation of raw Huangjing, but also helped to dry and store it. In ‘Supplement to Invaluable Prescription for Ready Reference’ by Simiao Sun, processing stages of Huangjing were refined by a drying method, also now known as double steaming, laying a foundation for the later method of nine steaming and nine drying (Sun SM 2014). Based on summarization of previous processing procedures, Meng Shen, in Dietetic Materia Medica, first put forward Huangjing processed by nine steaming and nine drying can lessen its discomfort and side effects (Meng S 2003).

### The Ming and Qing dynasties

Compared with pure steaming during the Tang and Song Dynasties, nine steaming and nine drying was added into many auxiliary materials, such as honey, wine, black beans, glutinous rice, vinegar, bile, and breast milk during the Ming and Qing Dynasties. For example, in the process of preparing Xixian, honey and wine were used as processing auxiliary materials. According to Diannan Materia Medica, *Asparagus cochin-chinensis* (Lour.) Merr. (Liliaceae) tuberous root (Tianmendong) adds wine during the process of nine steaming and nine drying, which can remove cold and stagnant properties of the drug due to the dispersing nature of wine (Lan M 2012). According to Bencao Shu, *Ligustri lucidi* fructus (Oleaceae) fruit (Nvzhenzi) were soaked in wine and then steamed with black beans to remove their cold properties. Bencao Huiyan points out that ‘raw Heshouwu is of cold qi, an astringent property and toxicity, while processed Heshouwu of mild qi and non-toxicity’ (Ni ZM 2005). The addition of black beans as auxiliary material not only leads the herb to the kidney channel, but also gets rid of its toxicity, ensuring TCM safety. Dahuang is a purgative agent and common drug in various books in Chinese medicine, involving raw Dahuang, Dahuang processed by nine steaming and nine drying, etc. Due to the dispersing effect of wine, addition of wine into nine steaming and nine drying of Dahuang not only reduces its cold property, but also relieves its purgative effect (Zhang R et al. 2021). In a word, during nine steaming and nine drying of TCM, addition of various auxiliary materials can rectify drug properties for a better clinical effect. In Compendium of Materia Medica, Shizhen Li provided a supplement to this processing method, ‘pour material into water to take floating matter out, dry it in the sun, steam it after mixing it with wine from morning to night, spread out and dry it in the sun’ (Li SZ 2005). This was a detailed record of the production process of nine steaming and nine drying.

### The modern and contemporary time

The number of steaming and drying times, as well as the ingredients used, have changed with the development of history. During the period of Republic of China, the best way to prepare cooked Dihuang was ‘to steam it with nine steaming and nine drying and it was best to have a deep black heart’ (Cao BH 2004); The processing of Xixian involves ‘sprinkling wine and honey layer by layer, steaming and then exposing it nine times’ (Zhang SL 2013). Some TCM varieties still retain the method of nine steaming and nine drying, such as Dihuang processed by nine steaming and nine drying in Henan province. Due to the complicated and cumbersome operation process, time-consuming, labor-intensive, and low efficiency, coupled with the advancement of processing technology, the process can be significantly optimized through various means. Some TCM varieties no longer use this processing method, such as Nvzhenzi, which is commonly used in modern times for steaming or stewing with yellow wine and can be completed in one step. In modern times, the methods have been inherited and improved. For example, Jiuzhi Dahuang Wan evolved from the method of nine steaming and nine drying (Xu et al. 2021). Through in-depth research on the principle of nine steaming and nine drying, the rationality has been further proven, providing modern scientific basis for its application.

## Purposes of nine steaming and nine drying

### Transform drug properties and expand application

Modern research has shown that during the process of nine steaming and nine drying, the quality and content of TCM components have changed, which also affects the properties of the medicine (Lee et al. 2019; Kim et al. 2019; Guo et al. 2019). To meet various clinical needs, doctors of all dynasties processed Dihuang with different methods. Compared with raw Dihuang, Dihuang processed by nine steaming and nine drying transforms bitter and cold properties into mild and nourishing ones and show effects of tonifying essence of life and dispersing qi, nourishing yin and blood (Liu TT et al. 2022). Huangjing has multiple health benefits and is honored as the king of tonifying both blood and qi (Zhu et al. 2014; Li XL et al. 2023). Modern pharmacological studies focused on boosting immunity, blood sugar lowering, antiatherosclerosis, and anticancerous potential (Zhang SX et al. 2020; Gu et al. 2020; Li et al. 2020; Zhang SQ et al. 2021). Repeated steaming and drying increases the bioactivity *via* better efficacy of medicinal and food components and its properties are greatly changed into enriching yin and nourishing kidney as well as tonifying both qi and blood (Sun et al. 2023). This method not only gets rid of numbness of raw Huangjing, but also enhances its tonifying effect. Modern pharmacological experiments have proved that the antioxidant, anti-fatigue, antibacterial and immunological activities of Huangjing were enhanced by steaming and drying (Zhao YY, et al. 2023; Yang et al. 2020; Cui et al. 2018). Different processed products of Huangjing can improve the symptoms of Qi-Yin deficiency in rats. Four-fold processed Huangjing has better effect in increasing body mass and tail diameter of the rats with Qi-Yin deficiency, and it is better at regulating glycolipid metabolism in rats, while nine-fold processed Huangjing can improve the liver function of Qi-Yin deficiency rats more significantly (Ma et al. 2019).

### Rectify properties and reduce adverse reactions

Ancients discovered that if Huma is not well steamed, it may lead to hair loss; if Huma is processed by nine steaming and nine drying, hair loss can be avoided; raw Heshouwu is of cold qi, an astringent property and toxicity, while processed Heshouwu of mild qi and non-toxicity. Thus, nine steaming and nine drying can rectify properties and reduce adverse reactions of some herbs. In the course of long-term clinical practice, people found that processed Heshouwu can exert a wide range of clinical effects with less adverse effect, which is consistent with traditional Chinese medicine theory that appropriate processing methods can reduce the toxicity of medicinal materials, improve their efficacy, and alter their pharmacological effects (Lei et al. 2015; Li et al. 2017).

**Figure 1.**
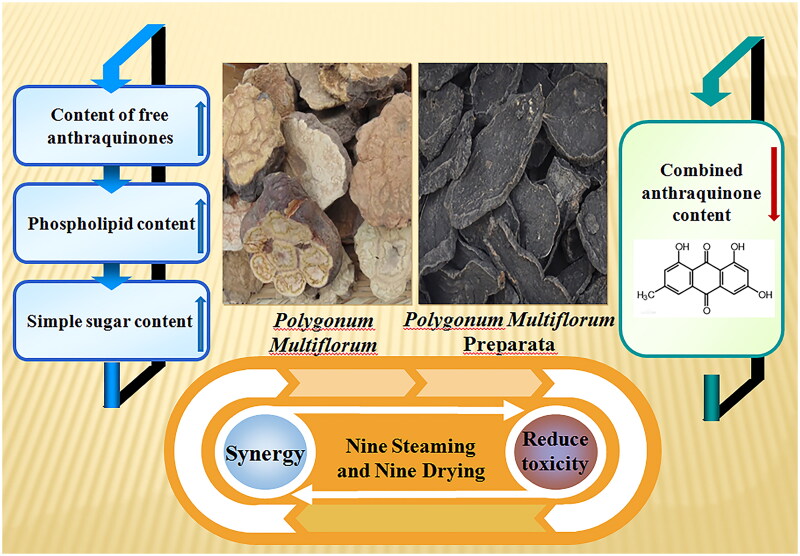
The purpose of nine steaming nine drying drugs - taking *polygonum multiflorum* as an example.

As one of the oldest and best-known Chinese herbal medicines, Dahuang is of a cold property and bitter taste with strong purgative effect and a common drug for constipation (Yuan S et al. 2019; Qi et al. 2021; Wang H et al. 2023). It is usually used as an effective, short-lived and painless cathartic for the treatment of purging accumulation, cooling blood, and draining damp-heat (Khiveh et al. 2017; Yang et al. 2018; Chen et al. 2019). Clinically, raw Dahuang is a severe laxative and is only recommended for small doses and short periods of time (Wang and Ren 2009). Modern research indicates that by nine steaming and nine drying, the purging effect of Dahuang can be relieved, while its effects of promoting blood circulation and removing blood stasis can be enhanced, leading to a better clinical effect (Gao et al. 2013; Zhu et al. 2017). Research has found that compared to raw Dahuang, the purgative effect of cooked Dahuang is reduced by 90%, which can make the action time longer and safer for clinical application (Lu HX 2012). According to both ancient literature and modern research, properties of Huangjing, Dahuang, and Dihuang are greatly changed by nine steaming and nine drying, representing typical cases of property change of TCM. The purpose of preparing TCM with nine steaming and nine drying is shown in Figure 1.

## Influences of nine steaming and nine drying on chemical composition

Nine steaming and nine drying are widely used to improve the quality, reduce toxicity and improve efficacy of TCM. The principle is to promote a series of changes in the chemical composition of medicinal materials through the synergistic effect of heating treatment and natural weathering. In the process of multiple steaming and drying, the type and content of chemical components will change to a certain extent, including hydrolysis, oxidation, reduction and other reactions, so as to change the chemical composition of medicinal materials, improve the efficacy and stability. In addition, the toxic components of some Chinese medicinal materials will be destroyed or transformed, so as to reduce toxicity and improve the safety of clinical drugs. In the process of multiple drying, the drug is exposed to nature, which helps the microbial metabolism of the drug surface, adjust the water content, and improve the quality and stability of the drug. For example, during the process of steaming and drying, the aglycone and isomer of Dihuang and Huangjing increased, while large molecular components are decomposed to form small molecular components that are easy to be absorbed by the human body (Li et al. 2019; Zhao LX et al. 2023). In this process, polysaccharides were hydrolyzed to oligosaccharides and monosaccharides, which are also considered to be important substances for improving immunity (Wang Y et al. 2023).

Sesame seeds contain more than 50% oil and 20% proteins (Kanu PJ 2011; Dutta et al. 2022). It is also rich in carbohydrates, minerals, fat soluble vitamins, and some secondary metabolites, such as flavonoids, saponins, and phenolic compounds (Khademian et al. 2019; Wan et al. 2023). Sesame oil also contains over 80% linoleic acid and linolenic acid. Due to the presence of lignans such as sesamin, sesamol, and sesamolin, sesame oil is more resistant to oxidative changes than other vegetable oils (Shi et al. 2017; Ardakani et al. 2023). According to research findings, the lignans in sesame seeds have special health benefits, such as preventing inflammation, low cholesterol, cancer, and coronary artery disease (Zhang et al. 2022). However, some antinutrients also exist in unprocessed sesame, such as phytate and oxalate. These ingredients and the hard shell of sesame seeds affect human digestion and absorption (Escamilla-Silva et al. 2003). Therefore, sesame seeds need to undergo various processes such as baking, frying, steaming, and sun drying before consumption (Zhang et al. 2023). Steaming and drying can cause serious damage to the shell of black sesame, deepen its color, increase its oil yield, decrease its protein content, and reduce the relative content of monounsaturated fatty acids (MUFAs) and polyunsaturated fatty acids (PUFAs). The total relative content of aldehydes in steamed sesame is significantly higher than that in raw sesame, while the total relative content of hydrocarbons and esters is significantly reduced. Principal component analysis shows that the number of cycles has a certain impact on these variables of sesame (Pi et al. 2022). Black sesame processed by nine steaming and nine drying contained far more amino acids than raw black sesame (Su et al. 2018). As a type of functional material, amino acids are not only an indicator to evaluate TCM, but also display certain pharmacological activity.

Huangjing is widely consumed as medicine-homology-food in Asia for its tonic effect (Pan et al. 2020). Modern pharmacological studies have shown that Huangjing have significant pharmacological activity, which are indispensable to biological activities and pharmacological effects, such as their immune enhancing effect (Li L et al. 2018), antibacterial effect (Debnath et al. 2013), anti-inflammatory effect (Zhang et al. 2015), potential anticancer effect (Yang et al. 2015). Huangjing contains compounds such as polysaccharides (Liu et al. 2007; Sun et al. 2020), steroidal saponins (Tang et al. 2019), and alkaloids (Sun et al. 2005). Through modern research, polysaccharide is considered to be the main biologically active components. (Zhao et al. 2019; Wang et al. 2020; Li X et al. 2021). In addition, raw Huangjing also has a large amount of mucus, which has a significant stimulating effect on the throat. In order to avoid throat irritation and enhance curative effects, Huangjing is traditionally processed by steaming and drying until the herbs become black, soft and sweet (Nie et al. 2023; Qin et al. 2020). Steam treatment changed the molecular weight, monosaccharide composition, and particle size distribution of polysaccharides in Huangjing (Xu et al. 2022). Undergoing molecular degradation, aggregation, and depolymerization, the structure of polysaccharides with different steaming and drying times showed significant changes, and the relative molecular weight of polysaccharides significantly decreased. The content of short chain fatty acids, including propionic acid, isobutyric acid, valeric acid, and isovaleric acid, in the feces of mice in the 6-steam 6-sun dried Huangjing group and the 9-steam 9-sun dried Huangjing group were significantly increased, which also had a good effect on regulating and improving the abundance and diversity of microbial communities (Su L-L et al. 2023). In addition, after repeated steaming and drying of the herb, its ethanol extracts, water extracts, reducing sugar and free amino acids increased. The processed Huangjing has a significantly enhanced tonifying effect, it could tonify spleen and moisten lung, warm and invigorate kidney yang (Zhu et al. 2022). What’s more, as it’s easy for TCM with lots of mucilaginous substances to get mildewed because of moisture, nine steaming and nine drying contributes to storage of the herbs.

**Figure 2.**
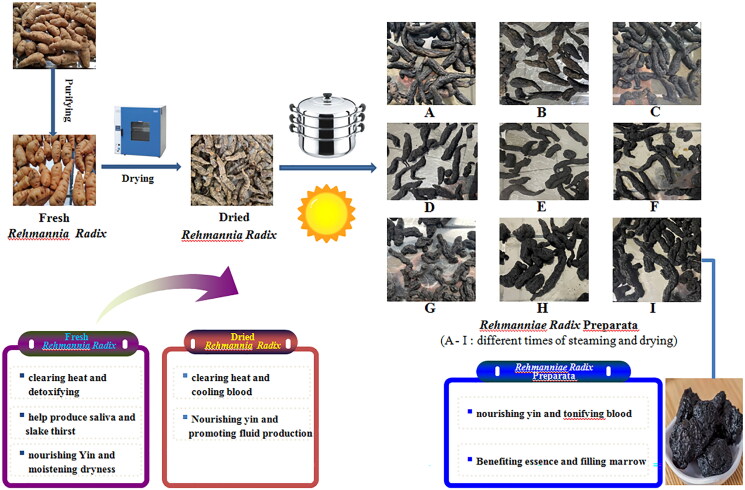
The characteristics and pharmacological changes of *Rehmanniae Radix* prepared by nine steaming and nine drying.

Dihuang is a widely used TCM and was listed as a ‘top grade’ herb in China (Zhang et al. 1993; Liu Y et al. 2022). According to traditional uses in China, Dihuang has the activity of nourishing Yin and tonifying the kidney (Kim et al. 1999). Modern research has found that Dihuang has a wide range of clinical activities, including hemostasis, promoting blood clotting, strengthening the heart, diuresis, hypoglycemic and anti-inflammatory activities, indicating that it has various pharmacological effects and phytochemical compositions (Yuan YY et al. 2019; Zhu et al. 2022). According to books on Chinese herbal medicine during all dynasties, there are many methods for processing Dihuang, such as steaming, processing with wine, processing with ginger juice, processing with other TCM and nine steaming and nine drying etc. (Liu et al. 2023). Now Dihuang can be divided into three medicinal types, namely fresh Dihuang, dry Dihuang, and prepared Dihuang. Big chemical change had occurred after the processing of Dihuang (He et al. 2014). Fresh and dry roots nourish yin, reduce blood and heat, increase saliva flow, and prepared Dihuang is mainly used to treat yang deficiency, which is beneficial for nutrition and improving bone marrow (Han et al. 2015; Wang HN et al. 2019). In the past few decades, more than 200 compounds were isolated, including iridoid, ionic ketone, phenylethanoid glycoside and so on (Zhang et al. 2008; Chen et al. 2021; Thu et al. 2021; Thu et al. 2022). During the processing, there were quantitative and qualitative changes in the components of Dihuang. Modern research indicates that by nine steaming and nine drying, raw Dihuang is transformed from reddish brown into pitch-black and prepared Dihuang contains much more polysaccharides than raw Dihuang. Clinical experience has shown that prepared Dihuang has almost completely lost its effectiveness in cooling blood and resolving blood stasis, mainly playing the role of nourishing blood and tonifying the body (Gong et al. 2021). In addition, research has confirmed that the antithrombotic effect of dry Dihuang is significantly better than that of prepared Dihuang (Gong et al. 2019). After repeated evaporation, various chemical components such as iridoid glycosides, phenylethanolamine glycosides, monosaccharides, and oligosaccharides have been reduced to varying degrees (Zhou et al. 2016; Xue et al. 2018). Some scholars also found that during the process of nine steaming and nine drying, the content of motherwort glycoside and catalpol in Dihuang increased, both of which are the material basis for nourishing yin and nourishing blood in prepared Dihuang (Meng et al. 2016). However, as the number of times of steaming and drying increases, the glycosides and catalpol of Dihuang gradually decrease, which may be due to their poor thermal stability. Reducing sugar first increased and then decreased and it reached a maximum value in the seventh time. With the increasing times of steaming and drying, verbascosides, sucrose, raffinose and stachyose gradually decreased, martynosides A and D slightly increased, and fructose, glucose and mannotriose significantly increased (Shu et al. 2023). Lu et al. (2022) showed that most glycosides and amino acids of processed Dihuang were degraded, the processed herb turned black and produced new ingredients, including 5-hydroxymethylfurfural and reducing sugar, indicating that both chemical composition and contents of Dihuang were changed by repeated steaming and drying. The characteristics and pharmacological changes of cooked Dihuang prepared by nine steaming and nine sun drying are shown in Figure 2.

## Influences of nine steaming and nine drying on pharmacological activity

### Antioxidant activity

The free radical scavenging activity of Huangjing significantly increases after steaming and gradually increases with the increasing time of processing (Li QY et al. 2021). Cellular experiments showed Huangjing processed with steaming and drying can significantly improve memory impairment in d-galactose damaged mice, with steaming and drying five times having the best effect and it showed the best effect in preventing cell death and synaptic damage in mice with d-galactose injury. In addition, in mice damaged by d-galactose, Huangjing steamed and dried five times increased the expression of antioxidant stress related proteins and decreased the expression of inflammatory related proteins (Bian et al. 2022). Researchers have verified that the extract from Heshouwu processed by nine steaming and nine drying (Zhishouwu) presented a higher 1,1-diphenyl-2-picrylhydrazyl (DPPH) radical scavenging activity than the extract from raw Heshouwu, with IC_50_ values of 0.43 mg/mL and 2.9 mg/mL, respectively (*p* < 0.05) (Liu et al. 2011).

### Regulate immune function

Polysaccharides in both Huangjing and Dihuang by nine steaming and nine drying can significantly improve the immune function of the body. Recent studies have shown that polysaccharides of Huangjing can significantly stimulate serum immunoglobulins, improve the proliferation of peripheral blood T lymphocytes, and up regulate the expression of IL-2, IL-6, and IFN-γ genes. The expression of interferon gene shows significant immune regulatory activity (Shu et al. 2021). Steam treatment changed the molecular weight, monosaccharide composition, and particle size distribution of polysaccharides in Huangjing (Wu et al. 2022; Bian et al. 2023). Polysaccharides in Dihuang can activate immune cells *in vitro* and can serve as effective adjunctive molecules for cancer immunotherapy (Fan QL et al. 2020; Xu et al. 2017). A recent study finds that polysaccharides in prepared Dihuang showed no cytotoxic effects on and significantly promoted the phagocytic activity of RAW264.7 cells. They dose-dependently improved lysozyme activity and stimulated the production of TNF-α and IL-6 by RAW264.7 cells, but attenuated the secretion of lysozymes, TNF-α, IL-6, IL-1β, and nitric oxide by lipopolysaccharide-induced RAW264.7 cells. The studies suggest that polysaccharide of prepared Dihuang is a valuable source with immunomodulating (Zhou et al. 2021). Meanwhile, the polysaccharide content of Dihuang processed by nine steaming and nine drying is higher than that in raw Dihuang. This is because there are a large amount of glycosides in raw Dihuang, high temperature and prolonged heating can cause its degradation to produce monosaccharides such as glucose and rhamnose, leading to an increase in polysaccharide content (Zhang et al. 2019).

### Regulate blood glucose

With the in-depth study of the nine steaming and nine drying methods, people have found that TCM processed by this method shows a good effect upon regulating blood glucose. By exploring potential active sites, inhibition of glucosidase activity was tested on different extraction sites of crude and steam treated Huangjing. The result shows that that the active ingredient in the ethyl acetate phase of the steamed product has a strong effect on α-glucose (Chen et al. 2015; Luo et al. 2022). When Huangjing was processed using the traditional technology of nine steaming and nine drying, 70% ethanol extracts exhibited the relief of glycolipid metabolism abnormalities in type 2 diabetic mice (Wang WX et al. 2019).

### Anticancer effects

Scientific studies have shown that processed Huangjing and processed Dihuang obtained by nine steaming and nine drying have anticancer activity (Zhao LX, et al. 2023; Li X et al. 2018). Processed Huangjing can significantly inhibit the proliferation, invasion and migration of Eca 109 cells and promote apoptosis (Zhou et al. 2019). Polysaccharides of processed Huangjing can exerted an immune-enhancing effects on lung cancer by activating TLR-4 receptor and the downstream MAPK/NF-κB signaling pathway (Long et al. 2018). Its function is related to the expression of related apoptotic proteins. And it can also increase the level of immune factors, stimulate the body’s immune response, and promote the body’s own antitumor response (Li X et al. 2018). A recent study found that polysaccharides of processed Dihuang can reverse drug resistance in tumor cells. It can promote autophagy, induce apoptosis and inhibit proliferation on docetaxel resistant prostate cancer cell line DU145, and its mechanism may be related to the regulation of mTOR signaling pathway (Xia et al. 2022).

### Other pharmacological activities

Processed Dihuang is widely used as primal medicine in Chinese herbal formula for the treatment of Alzheimer ‘s disease (AD). The researchers have initially revealed the pharmacological mechanism, indicated that the compounds in processed Dihuang have binding ability to INSR protein and potential multiple synergistic effects. Processed Dihuang ameliorates cognitive dysfunction and brain histopathological changes in AD mice. The mechanism of ameliorating AD may be related to the regulation of INSR/IRS-1/AKT/GSK-3β signaling pathway and intestinal microbiota (Su YF et al. 2023). Another research suggests that catalpol of processed Dihuang exhibits significant anti-diabetic bioactivity, and thus it has attracted increasing research attention for its potential use in treating diabetic nephropathy (Fu et al. 2023). Treatment with catalpol significantly improved renal function in diabetic nephropathy animal models by restoring serum creatinine and blood urea nitrogen levels, reducing proteinuria and fasting blood glucose, improving kidney indices, and alleviating renal pathological changes in the animal models (Yang C et al. 2020; Wang et al. 2021). Zhishouwu can improve liver mitochondrial function and alleviate glucolipid metabolic disorders by regulating mitochondrial metabolic pathways. Therefore, the application of Zhishouw may be a promising mitochondrial regulatory factor/nutrient for alleviating glucolipid metabolic disorders (GLMD) related diseases (Yang et al. 2021). In addition, Zhishouwu can also delay skin aging by regulating mitochondrial autophagy and removing reactive oxygen species (ROS) (Liu et al. 2021). This can further explain the function of Heshouwu and lay the foundation for mitochondrial autophagy as a target for delaying skin aging.

## Summary and prospect

Though chemical constitution of some TCM by nine steaming and nine drying has been determined in modern research, no in-depth study on the mechanism of nine steaming and nine drying has been seen so far. For instance, ‘being steamed to complete black from inside out with sweet sour taste without bitterness’ is a standard for prepared Dihuang, which is vague and not technological. Hence, it is necessary to establish the processing standards and requirements of nine steaming and nine drying products, and further research on the processing mechanism is needed.

### Literature research on times of steaming and drying

Though nine steaming and nine drying is recorded in many ancient books of Chinese medicine, the ancient ‘nine’ has many meanings, some indicate the exact number, some indicate the repeated behavior, and some refer to a long time because of the sound with ‘long’ (Liu J et al. 2022). Also, it may be related to ancient Chinese understanding of nine, as Seeking Truth from the Original Grass says, ‘…steamed and dried nine times (numbers ends with nine)’ (Huang GX 1997). According to books related, Huma should be fully steamed and requires 30 times of steaming for ‘raw Huma leads to hair loss’. Prepared Dihuang should be ‘completely dark and as sweet as maltose’. *Atractylodes macrocephala* Koidz. (Compositae) root (Baizhu) can be steamed ten times until ‘hands can feel grease from the herb’. Nvzhenzi should be ‘steamed, dried and moistened seven times respectively’. *Achyranthes bidentata* Blume. (Amarantnaceae) root (Niuxi) ‘should be steamed dozens of times after mixing with Huangjing juice and wine’. The specific processing details vary depending on the type of medicinal material, and the main purpose of this method is to control the medicinal properties of the medicinal material or improve its therapeutic effect (Jiang et al. 2019; Liao et al. 2022). Therefore, processing times of drugs by steaming and drying remain to be discussed and are worthy of further research.

### Establish objective evaluation index

Traditional processing indicators are not objective and often rely on subjective descriptions, making it difficult to accurately reflect the characteristics of changes in the decoction pieces during the process of nine steaming and nine drying. In ancient times, the processing methods of Huangjing and Dihuang were mostly based on the standard of ‘black as paint, sweet as malt’ to determine the processing endpoint. Color, luster, and form were used as evaluation criteria to achieve a black, moist, and sweet taste. It is easy to cause problems such as uneven processing degree and uneven quality of decoction pieces, which affects clinical efficacy. Therefore, one direction that needs to be focused on is to use modern technological methods to quantify the process of nine steaming and nine drying, to make the evaluation indicators objective and fair. As an emerging artificial intelligence sensory technology based on simulating human senses, electronic eyes, electronic tongues, electronic noses, etc. can objectively characterize changes in the color, odor, taste, and other aspects of Chinese herbal medicine slices, especially accurately distinguish small differences between different processing methods. It provides technical support for the digital expression of traditional evaluation indicators for TCM decoction pieces. Applying it to the analysis of changes in traditional Chinese medicine processing can not only transform traditional identification experience into objective data, but also evaluate the quality of TCM processing. In the future research work, the processing technology of nine steaming and nine drying can be discussed through the change of main chemical components and the objective characterization method of intelligent sensory analysis technology.

### Further study on processing mechanism

At present, most of the research on the mechanism of nine steaming and nine drying processing adopts a single chemical evaluation or pharmacological action, and there is a lack of research on the processing mechanism and metabolism, which cannot scientifically diagnose and interpret its complex system. The therapeutic effect of TCM has the characteristics of multiple targets and pathways. Computer simulation methods of network pharmacology can be used to speculate on the potential targets and mechanisms of action of nine steaming and nine drying, and then verify them through experiments *in vivo in* subsequent research. HPLC-ESI/MS and other advanced technology should be used to determine the qualitative and quantitative characteristics of the components before and after steaming and drying. It is necessary to explore the transformation mechanism of ingredients in the process of steaming and drying, clarify the differences in absorption and metabolism of such ingredients *in vivo*, reveal the material basis of steaming and drying products, and clarify the processing mechanism from the perspective of transformation and absorption metabolism of active ingredients.

### Attach importance to research on processing adjuvants

Processing adjuvants are used in nine steaming and nine drying frequently, such as rice wine, sand kernel, soybean and other auxiliary materials. According to Chinese Pharmacopoeia (2020 edition) and the processing standards of various provinces in China, the processing method of Heshouwu is mainly to add black bean juice and mix it well before steaming. The effects of adjuvants in the process of repeated steaming and drying can be regarded as an important research direction. But there are few studies on the changes of active/toxic components of processing adjuvants or other drugs, and the studies on the changes of adjuvants on the trend of channel distributions are relatively scarce. Because of the lack of scientific and unified processing technology and standard of adjuvants, the quality and clinical application effect of decoction pieces are affected, and this is also the focus of the later research.
